# Preschool teachers’ intention to organize physical activity: an application of the norm activation model

**DOI:** 10.3389/fpsyg.2026.1971172

**Published:** 2026-10-01

**Authors:** Zeying Miao, Jiantuan Huang, Rui Xue, Yinjian Qin

**Affiliations:** 1 School of Education, Beijing Sport University, Beijing, China; 2 College of Physical Education and Health Science, Guangxi Minzu University, Nanning, China

**Keywords:** behavioral intention, early childhood education, norm activation model, physical activity promotion, preschool teachers

## Abstract

**Background:**

Preschool teachers play an important role in providing children with physical activity opportunities. Existing research has focused mainly on teachers’ professional competence and organizational environments, while responsibility-related normative correlates of their intention to organize physical activity remain less understood. Guided by the Norm Activation Model (NAM), this study examined associations among awareness of consequences, ascription of responsibility, personal norms, and intention to organize physical activity.

**Methods:**

A cross-sectional survey was conducted among 582 in-service preschool teachers in China using convenience sampling. Confirmatory factor analysis and structural equation modeling tested the measurement and structural models. Direct, indirect, and total associations were examined using bias-corrected bootstrapping with 5,000 resamples.

**Results:**

The retained partial mediation model showed good fit (*χ2* = 119.984, df = 115, *p* = 0.357, RMSEA = 0.009, 90% CI [0.000, 0.023]). Awareness of consequences was positively associated with ascription of responsibility (*β* = 0.522), which was positively associated with personal norms (*β* = 0.617); personal norms were positively associated with behavioral intention (*β* = 0.727; all *p* < 0.001). Awareness of consequences was also directly associated with behavioral intention (*β* = 0.173, *p* < 0.001). The standardized sequential indirect association ascription of responsibility and personal norms was 0.234 (95% CI [0.188, 0.282]).

**Conclusion:**

Preschool teachers’ intention to organize physical activity was associated with awareness of the developmental consequences of insufficient physical activity, perceived responsibility for addressing them, and a personal obligation to act. This pattern was consistent with NAM. Promotion efforts should connect teachers’ understanding of the developmental value of physical activity with clear professional responsibilities and the practical and organizational support needed to provide regular physical activity opportunities in preschool settings.

## 1 Introduction

Physical activity during early childhood plays an important role in children’s physical health, motor competence, cognitive functioning, and psychosocial wellbeing (Carson et al., 2017; Hinkley et al., 2014; Zeng et al., 2017). Adequate physical activity during the preschool years not only supports children’s current physical development and motor skill acquisition but may also contribute to the establishment of health-related behavioral patterns that persist into later childhood (Carson et al., 2017; Jones et al., 2013). In preschool settings, these developmental benefits also give physical activity clear educational significance, as regular opportunities for movement form part of children’s everyday learning and development. Accordingly, international guidelines recommend that preschool-aged children accumulate sufficient physical activity throughout the day while minimizing prolonged sedentary time (World Health Organization, 2019). Nevertheless, many young children remain insufficiently active in their daily lives. Evidence from preschools and other early childhood education and care settings indicates that they spend a substantial proportion of their time sedentary and engage in relatively limited amounts of moderate-to-vigorous physical activity (O’Brien et al., 2018). Because young children’s access to physical activity is strongly shaped by the time, space, support, and activity opportunities provided by adults, efforts to promote their physical activity should extend beyond a sole focus on children themselves and consider how such opportunities are organized and implemented in preschool settings (Martin et al., 2022; Tonge et al., 2024; Wright et al., 2024).

Preschool teachers play a central role in planning and providing opportunities for children’s physical activity within everyday preschool routines. Through activity scheduling and planning, instructional support, encouragement, active participation, and decisions about planned physical activities and indoor and outdoor play, teachers help shape both the availability of these opportunities and the ways in which children engage with them (Tonge et al., 2024; Wright et al., 2024). Yet teachers’ capacity to provide such opportunities is also shaped by practical constraints, including limited time and space, workload and staffing pressures, safety concerns, and competing curricular priorities (Copeland et al., 2012; Jerebine et al., 2024). Under such conditions, physical activities may be shortened, simplified, postponed, or deprioritized in favor of other educational tasks. Although environmental and organizational conditions are undoubtedly important, they do not fully determine how teachers respond to these constraints. Whether physical activity is incorporated into everyday practice may also be associated with educators’ motivation and intentions, including the extent to which they prioritize physical activity amid competing educational demands (Copeland et al., 2012; Gagné and Harnois, 2014; Jerebine et al., 2024). Given that behavioral intention is generally regarded as an important proximal antecedent of subsequent behavior, and that educators’ psychosocial characteristics have been linked to young children’s physical activity in childcare settings, understanding the factors associated with preschool teachers’ intention to organize physical activity is an important step toward explaining variation in their organizational practices (Ajzen, 1991; Gagné and Harnois, 2013; Sheeran, 2002).

Existing explanations of preschool teachers’ physical activity-promoting practices have emphasized professional knowledge, competence, confidence, time, space, training, staffing, and organizational support (Asada et al., 2023; Bourke et al., 2024; Bruijns et al., 2021; Copeland et al., 2012; Jerebine et al., 2024; Leung et al., 2024; Martin et al., 2022; Tonge et al., 2024). These factors address whether teachers are capable of organizing physical activity and whether their workplaces enable it. The Norm Activation Model (NAM) adds a theoretically distinct responsibility-related dimension: whether teachers recognize the consequences of insufficient activity, regard addressing them as their own responsibility, and report a personal obligation to act. Accordingly, the present study applies NAM to better understand preschool teachers’ intention to organize physical activity by examining a responsibility-related normative dimension that has received limited attention in previous research.

NAM provides a focused framework for examining the responsibility-related normative factors associated with preschool teachers’ intention to organize physical activity. In its commonly used sequential formulation, awareness of the developmental consequences of insufficient physical activity is associated with ascription of responsibility, which is in turn associated with personal norms and behavioral intention (Schwartz, 1977; de Groot and Steg, 2009). Whereas previous research has primarily emphasized teachers’ professional competence and organizational conditions, the responsibility-related pathway underlying their intention to organize physical activity has received limited attention. Accordingly, the present study examines the associations among awareness of consequences, ascription of responsibility, personal norms, and behavioral intention in this specific educational context.

## 2 Theoretical background and hypotheses

### 2.1 Organizing physical activity as a responsibility-oriented behavior in preschool settings

In preschool settings, organizing physical activity forms part of teachers’ broader educational responsibility for supporting children’s development. Regular opportunities for movement contribute to children’s physical health, motor competence, cognitive functioning, and psychosocial wellbeing, while children’s access to these opportunities is shaped in part by how physical activity is planned and incorporated into daily routines.

Preschool teachers are therefore directly involved in shaping children’s opportunities for physical activity through decisions about scheduling, activity content, instructional support, and indoor and outdoor movement. When such opportunities are insufficient, the developmental consequences of inadequate physical activity become relevant to how teachers understand their own role and responsibility. From this perspective, organizing physical activity can be examined as a responsibility-oriented behavior, providing a basis for applying the Norm Activation Model to teachers’ intention to organize physical activity (Schwartz, 1977; Steg and de Groot, 2010).

Professional-role expectations, ascription of responsibility, and personal norms are conceptually distinct. Professional-role expectations refer to duties prescribed by curricula, institutional policies, occupational standards, or other external sources. Ascription of responsibility concerns whether teachers regard themselves as accountable for children’s opportunities for physical activity and the potential consequences of insufficient activity, whereas personal norms refer to a personally experienced obligation to act.

Organizing physical activity in preschool settings involves responsibility-related judgments within a broader professional and institutional context. Teachers’ decisions about when, where, and how such activities are provided are shaped by curriculum expectations and institutional arrangements concerning scheduling, space, staffing, safety, and administrative authority. Against this background, NAM offers a focused framework for examining how awareness of consequences, ascription of responsibility, and personal norms are associated with preschool teachers’ intention to organize physical activity.

### 2.2 Norm activation model

NAM, originally proposed by Schwartz, has been widely used to explain prosocial and responsibility-oriented behavior (Schwartz, 1977). Rather than focusing primarily on external constraints or instrumental considerations, the model emphasizes the role of personally endorsed norms in relation to behavioral intention.

According to NAM, greater awareness of the developmental consequences of insufficient physical activity is associated with stronger ascription of responsibility. When teachers regard themselves as responsible for addressing these consequences, they may experience a stronger personal obligation to act, which in turn is associated with a stronger intention to organize physical activity (Schwartz, 1977; Steg and de Groot, 2010). This framework is particularly relevant in contexts where behavior carries implicit expectations of responsibility. As discussed above, organizing physical activity in preschool settings involves not only instructional considerations but also concern for children’s development and everyday wellbeing. In such contexts, teachers’ decisions may be shaped less by situational convenience and more by how they interpret potential consequences and their own responsibility.

Taken together, NAM provides a theoretical basis for examining how awareness of consequences, ascription of responsibility, and personal norms are related to preschool teachers’ intention to organize physical activity.

### 2.3 Hypothesis development

Within NAM, awareness of consequences is theorized as an antecedent of responsibility attribution. When individuals recognize that insufficient or inappropriate action may be associated with negative outcomes, they may be more likely to reflect on their own role in relation to those consequences (Schwartz, 1977; Steg and de Groot, 2010). In preschool settings, teachers who report greater awareness that insufficient physical activity may hinder children’s physical, motor, and socio-emotional development may also be more likely to regard themselves as responsible for ensuring adequate opportunities for activity. Accordingly, awareness of consequences is expected to be positively associated with ascription of responsibility (Schwartz, 1977; Steg and de Groot, 2010).

*H1*: Awareness of consequences is positively associated with ascription of responsibility.

Ascription of responsibility represents the extent to which individuals perceive themselves as accountable for particular outcomes. Within NAM, this perception is theorized as a condition associated with the activation or salience of personal norms (Schwartz, 1977; Steg and de Groot, 2010). When individuals acknowledge personal responsibility, they may also report a stronger sense of moral obligation regarding what they ought to do (Schwartz, 1977). In the context of preschool education, teachers who more strongly attribute responsibility to themselves are expected to report stronger personal norms regarding the organization of physical activity. Therefore, ascription of responsibility is expected to be positively associated with personal norms (Schwartz, 1977; Steg and de Groot, 2010).

*H2*: Ascription of responsibility is positively associated with personal norms.

Personal norms refer to personally experienced moral obligations related to individual behavior (Schwartz, 1977). Compared with general attitudes or preferences, personal norms reflect a sense of duty rather than an evaluation of the behavior. In educational contexts, teachers who report a stronger sense of obligation toward children’s development may also report a stronger intention to organize physical activity, even when practical constraints such as limited time or resources are present. Thus, personal norms are expected to be positively associated with behavioral intention (Schwartz, 1977; Steg and de Groot, 2010).

*H3*: Personal norms are positively associated with preschool teachers’ intention to organize physical activity.

NAM further proposes that awareness of consequences, ascription of responsibility, personal norms, and behavioral intention are linked through a sequential normative process (Schwartz, 1977; Steg and de Groot, 2010). In this process, awareness of the consequences associated with insufficient physical activity provides the basis for teachers to consider their own role in addressing those consequences. When teachers attribute greater responsibility to themselves, this sense of responsibility is more likely to be reflected in a stronger personal obligation to provide children with adequate opportunities for physical activity. Ascription of responsibility may therefore account for part of the association between awareness of consequences and personal norms.

*H4*: Ascription of responsibility mediates the association between awareness of consequences and personal norms.

Personal norms further connect responsibility judgments with behavioral intention. When teachers view the promotion of children’s physical activity as a responsibility they personally ought to fulfill, this obligation may become more closely associated with their intention to organize physical activity in daily educational practice. Personal norms may therefore represent an intermediate mechanism through which ascription of responsibility is associated with behavioral intention.

*H5*: Personal norms mediate the association between ascription of responsibility and behavioral intention.

These relationships together form the sequential pathway proposed in the present study. Greater awareness of the developmental consequences of insufficient physical activity may be associated with stronger ascription of responsibility, which may in turn be associated with stronger personal norms and, subsequently, greater intention to organize physical activity. This sequence provides the theoretical basis for examining the combined indirect association of awareness of consequences with behavioral intention through ascription of responsibility and personal norms.

*H6*: Ascription of responsibility and personal norms sequentially mediate the association between awareness of consequences and behavioral intention.

Given the cross-sectional design of the study, the proposed mediation hypotheses are examined as indirect associations consistent with the theoretical ordering of NAM.

## 3 Methods

### 3.1 Participants and procedure

This cross-sectional online questionnaire survey was conducted from September to November 2025 among in-service preschool teachers from different types of kindergartens across multiple provinces and cities in China. Participants were recruited using convenience sampling, and the survey link was distributed through kindergarten contacts and online preschool teacher groups. Eligible participants were currently employed preschool teachers who were directly involved in children’s daily educational activities, including physical activity organization.

The questionnaire introduction described the research purpose, stated that responses would be used only for academic research and would be analyzed in aggregate, and asked participants to answer according to their actual work and genuine views. It did not request names, mobile phone numbers, kindergarten names, or other directly identifying information. A total of 718 questionnaires were collected; after 136 were excluded because of substantial missing data, evident patterned responding, or logically inconsistent answers, 582 valid responses remained (81.1%). Women comprised 91.1% of the sample, broadly consistent with the gender composition of China’s kindergarten teaching workforce (Ministry of Education of the People’s Republic of China, 2026). The largest group had 2–5 years of teaching experience (39.3%); 42.8% reported having studied content related to preschool physical education, and 38.7% worked in kindergartens employing at least one full-time physical education teacher (Table 1). The study protocol was reviewed and approved by the Sports Science Experiment Ethics Committee of Beijing Sport University (Approval No. 2026024H). All participants provided informed consent before completing the questionnaire.

**Table 1:** Participant and workplace characteristics (*N* = 582).

Characteristic	Category	*n* (%)
Gender	Male	52 (8.9)
	Female	530 (91.1)
Teaching experience	≤1 year	105 (18.0)
	2–5 years	229 (39.3)
	6–10 years	156 (26.8)
	≥11 years	92 (15.8)
Highest educational attainment	Technical secondary school or below	18 (3.1)
	Associate degree	108 (18.6)
	Bachelor’s degree	391 (67.2)
	Master’s degree or above	65 (11.2)
Type of kindergarten	Public	324 (55.7)
	Private	258 (44.3)
Previous study of preschool physical education-related content	Yes	249 (42.8)
	No	333 (57.2)
Kindergarten employing at least one full-time physical education teacher	Yes	225 (38.7)
	No	357 (61.3)

Values are presented as *n* (%). Percentages were calculated based on the 582 valid responses and rounded to one decimal place.

### 3.2 Measures

Prior to the formal survey, the questionnaire was administered to an independent pilot sample of 164 in-service preschool teachers. The pilot and formal surveys used the same 17 items and five-point response format, and no changes were made to the items between the two administrations. Analysis of the pilot data showed good preliminary internal consistency across the four constructs, with Cronbach’s α coefficients ranging from 0.824 to 0.871 and corrected item–total correlations ranging from 0.615 to 0.705.

The questionnaire was administered in Chinese and assessed four constructs derived from the Norm Activation Model (NAM): awareness of consequences (AC), ascription of responsibility (AR), personal norms (PN), and behavioral intention (BI) (Schwartz, 1977; de Groot and Steg, 2009; Steg and de Groot, 2010). It comprised 17 contextualized items—four AC, three AR, five PN, and five BI items—rated from 1 (completely disagree) to 5 (completely agree). AC assessed teachers’ recognition of the developmental consequences of insufficient physical activity; AR assessed the extent to which teachers attributed responsibility for addressing these consequences to themselves; PN assessed their personally experienced obligation to provide adequate physical activity opportunities; and BI assessed their intention to organize physical activity. These measures correspond to the respective consequence awareness, responsibility attribution, personal obligation, and behavioral intention components of the proposed NAM framework. Internal consistency in the formal sample was good for AC (α = 0.813), AR (α = 0.810), PN (α = 0.861), and BI (α = 0.817). Supplementary Table S1 presents the administered Chinese wording, English translations, response anchors, and behavioral target or time frame for each item.

### 3.3 Statistical analysis

All statistical analyses were conducted using R version 4.4.2 (R Foundation for Statistical Computing, Vienna, Austria) and IBM SPSS Amos 26.0 (IBM Corp., Armonk, NY, United States). R was used for data screening, descriptive statistics, correlation analyses, normality assessment, reliability and validity calculations, and the supplementary polychoric-correlation analysis. Amos was used for the primary analyses, including confirmatory factor analysis (CFA), structural equation modeling (SEM), model comparisons, and bootstrap estimation of direct and indirect associations. All statistical tests were two-tailed, with the significance level set at *p* < 0.05.

#### 3.3.1 Descriptive statistics, normality, and common method bias

Descriptive statistics were calculated for participants’ demographic characteristics and the primary study variables. Categorical variables were summarized using frequencies and percentages, whereas continuous variables were presented as means and standard deviations. Pearson correlation coefficients were subsequently calculated among awareness of consequences, ascription of responsibility, personal norms, and behavioral intention to provide an initial assessment of the linear associations among the study variables.

The distributional characteristics of the data were then examined. Univariate normality was assessed using skewness and kurtosis, with absolute skewness values below 3 and absolute kurtosis values below 10 considered acceptable (Kline, 2023). Multivariate normality was evaluated using Mardia’s multivariate kurtosis coefficient and its critical ratio (Mardia, 1970; Cain et al., 2017) to determine whether the data met the distributional assumptions for SEM.

Common method bias was initially assessed using Harman’s single-factor test. A single-factor CFA model was subsequently specified by loading all measurement items onto one latent factor, and its fit was compared with that of the hypothesized multidimensional measurement model (Podsakoff et al., 2003). A substantially poorer fit of the single-factor model relative to the multidimensional model was interpreted as indicating that common method bias was not a serious concern (Podsakoff et al., 2003; Fuller et al., 2016).

#### 3.3.2 Measurement model

CFA was performed using maximum likelihood (ML) estimation to examine the measurement structure of awareness of consequences, ascription of responsibility, personal norms, and behavioral intention. Overall model fit was evaluated using the chi-square to degrees-of-freedom ratio (*χ2*/df), comparative fit index (CFI), Tucker–Lewis index (TLI), root mean square error of approximation (RMSEA), and standardized root mean square residual (SRMR). Acceptable model fit was defined as *χ2*/df < 3, CFI and TLI ≥ 0.90, and RMSEA and SRMR ≤0.08 (Hu and Bentler, 1999; Schermelleh-Engel et al., 2003; Kline, 2023).

Following evaluation of overall model fit, measurement quality was examined. Internal consistency was assessed using Cronbach’s alpha and composite reliability (CR), with values ≥0.70 considered acceptable for both indices (Cronbach, 1951; Raykov, 1997; Nunnally and Bernstein, 1994). Convergent validity was evaluated using standardized factor loadings, CR, and average variance extracted (AVE), with acceptable values defined as ≥0.50, ≥0.70, and ≥0.50, respectively (Fornell and Larcker, 1981; Hair et al., 2019). Discriminant validity was assessed using the Fornell–Larcker criterion, whereby the square root of the AVE for each latent construct was required to exceed its correlations with the other latent constructs (Fornell and Larcker, 1981), and was further examined using the heterotrait–monotrait ratio of correlations (HTMT), with values below 0.90 considered acceptable (Henseler et al., 2015).

Competing measurement models included two three-factor models, one combining AR with PN and the other combining PN with BI; a two-factor model specifying AC as one factor and combining AR, PN, and BI as the other; and a one-factor model in which all items loaded onto a single factor. These models were compared with the hypothesized four-factor model.

#### 3.3.3 Structural model, indirect effects, and robustness analyses

After the measurement model demonstrated acceptable fit, reliability, and validity, a structural equation model was specified to examine the hypothesized relationships among awareness of consequences, ascription of responsibility, personal norms, and behavioral intention. The structural model was estimated using the same parameterization as the validated measurement model, and all structural paths were examined within a single integrated modeling framework. Hypotheses were evaluated using standardized path coefficients, critical ratios, and corresponding *p* values. A path was considered statistically significant when the absolute critical ratio exceeded 1.96 and *p* < 0.05.

The theoretically specified fully sequential mediation model (S1) was first examined. In this model, awareness of consequences predicted ascription of responsibility, ascription of responsibility predicted personal norms, and personal norms predicted behavioral intention, with no direct paths bypassing adjacent variables in the sequential chain. An expanded model (S2) was subsequently estimated by adding three theoretically plausible direct paths: awareness of consequences to personal norms, awareness of consequences to behavioral intention, and ascription of responsibility to behavioral intention. The two added direct paths that were nonsignificant in S2 were then constrained to zero, yielding a more parsimonious partial mediation model (S3) that retained the direct path from awareness of consequences to behavioral intention. Because S1, S2, and S3 were nested, differences in model fit were evaluated using chi-square difference tests. The final model was selected based on the chi-square difference tests, overall model fit, parsimony, the Akaike information criterion (AIC), the Bayesian information criterion (BIC), and theoretical consistency, with lower AIC and BIC values indicating a preferable model (Akaike, 1974; Schwarz, 1978; Kline, 2023).

Indirect effects were tested using bias-corrected bootstrap confidence intervals (CIs) based on 5,000 resamples. Specific indirect effects, the total indirect effect, and the total effect were estimated separately. Statistical significance was determined by whether the 95% CI for an indirect effect excluded zero (Cheung, 2007; Preacher and Hayes, 2008).

Several supplementary analyses were conducted to evaluate the stability and robustness of the model findings. First, theoretically plausible non-nested alternative models were estimated and compared using AIC and BIC, with lower values indicating a preferable balance between model fit and parsimony (Akaike, 1974; Schwarz, 1978). Second, a polychoric-correlation refit was conducted to examine whether the principal path estimates were robust to treating the five-point response categories as ordinal variables. Third, theoretically relevant demographic variables were included as covariates in the structural model. Changes in the principal path coefficients after adjustment were examined to evaluate the robustness and consistency of the primary findings.

## 4 Results

### 4.1 Preliminary analyses

Item-level skewness ranged from −0.903 to −0.202, and kurtosis ranged from −0.962 to −0.043. Mardia’s multivariate kurtosis was 327.256, with a critical ratio of 2.020, indicating a slight departure from multivariate normality. In Harman’s single-factor test, the first unrotated factor accounted for 38.08% of the total variance. The mean scores of the four constructs ranged from 3.402 to 4.018. All correlations were positive and statistically significant, ranging from 0.271 to 0.658 (all *p* < 0.01). The strongest correlation was observed between personal norms and behavioral intention (r = 0.658), followed by the correlation between ascription of responsibility and personal norms (r = 0.517). The correlation matrix is presented in Figure 1.

**Figure 1:**
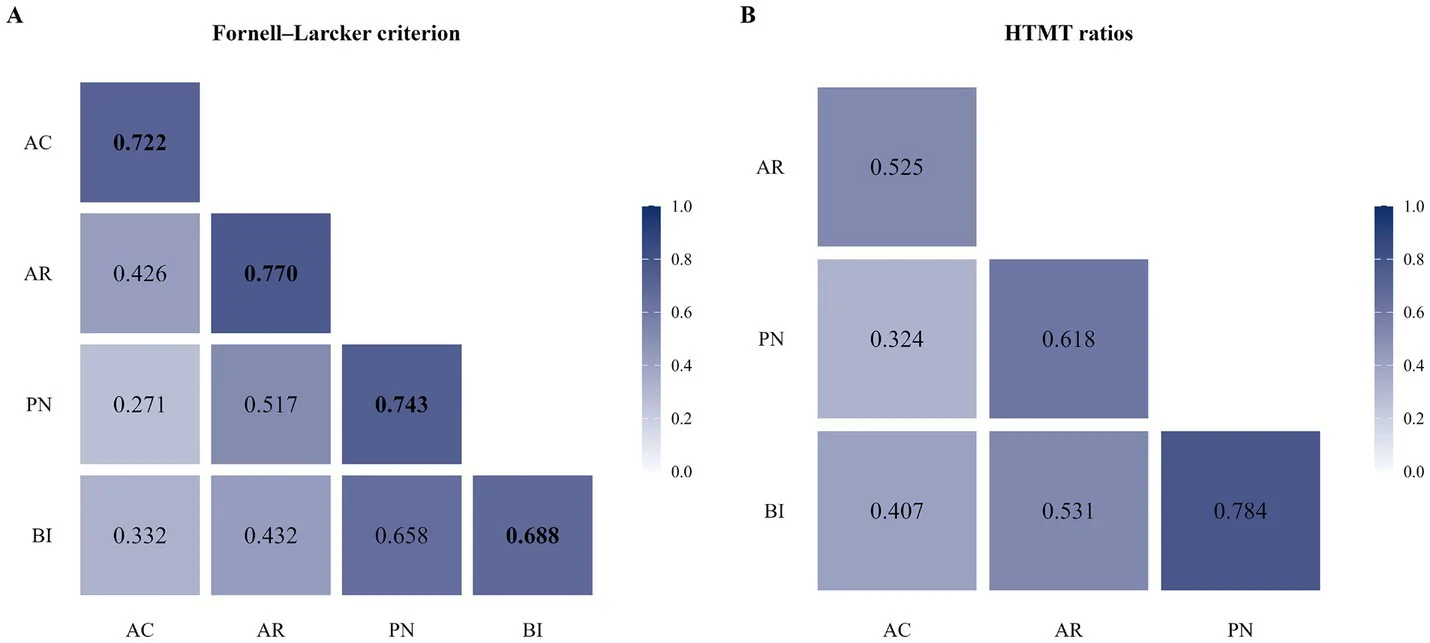
Discriminant validity assessment using the Fornell–Larcker criterion and HTMT ratios. Panel **(A)** presents the Fornell–Larcker criterion. Bold diagonal values are the square roots of average variance extracted (AVE), and values below the diagonal are Pearson correlation coefficients. Panel **(B)** presents heterotrait–monotrait (HTMT) ratios. Darker shading indicates larger values. All Pearson correlations were significant at *p* < 0.01. AC = awareness of consequences; AR = ascription of responsibility; PN = personal norms; BI = behavioral intention.

### 4.2 Measurement model

The hypothesized four-factor measurement model showed a good fit, *χ2* = 119.827, df = 113, *p* = 0.312, *χ2*/df = 1.060, CFI = 0.998, TLI = 0.998, RMSEA = 0.010, 90% CI [0.000, 0.024], and SRMR = 0.025. The four-factor model fit significantly better than the three-factor model combining AR and PN, Δ*χ2* = 376.090, Δdf = 3, *p* < 0.001, and the three-factor model combining PN and BI, Δ*χ2* = 188.991, Δdf = 3, *p* < 0.001. It also outperformed the one-factor and two-factor alternatives. The poorer fit of the one-factor model was consistent with Harman’s single-factor test, indicating that a single common factor did not account for the covariance among the measurement items (Table 2).

**Table 2:** Comparison of alternative measurement models.

Model	*χ2*	*df*	*χ2*/*df*	CFI	TLI	RMSEA	SRMR	AIC	BIC	Δ*χ2*	Δ*df*	*p*
One-factor model	1254.853	119	10.545	0.721	0.681	0.128	0.104	28217.260	28365.720	–	–	–
Two-factor model	685.287	118	5.808	0.860	0.839	0.091	0.071	27648.713	27801.540	569.566	1	<0.001
Three-factor model A (AC; AR + PN; BI)	495.917	116	4.275	0.907	0.890	0.075	0.063	27463.017	27624.576	189.370	2	<0.001
Three-factor model B (AC; AR; PN + BI)	308.818	116	2.662	0.953	0.944	0.053	0.042	27275.596	27437.156	376.469	2	<0.001
Four-factor model	119.827	113	1.060	0.998	0.998	0.010	0.025	27092.280	27266.938	188.991	3	<0.001

The one-factor model combined all 17 items into a single factor. The two-factor model specified AC as one factor and combined AR, PN, and BI as the second factor. Three-factor model A specified AC and BI as separate factors and combined AR with PN; Three-factor model B specified AC and AR as separate factors and combined PN with BI. The reported Δ*χ2* tests compare the two-factor model with the one-factor model, each three-factor model with the two-factor model, and the four-factor model with Three-factor model B. The four-factor model also fit significantly better than Three-factor model A, Δ*χ2* = 376.090, Δdf = 3, *p* < 0.001. No *χ2* difference test was conducted between models A and B because they were non-nested. AC = awareness of consequences; AR = ascription of responsibility; PN = personal norms; BI = behavioral intention; CFI = comparative fit index; TLI = Tucker–Lewis index; RMSEA = root mean square error of approximation; SRMR = standardized root mean square residual; AIC = Akaike information criterion; BIC = Bayesian information criterion.

All standardized factor loadings were significant and ranged from 0.662 to 0.852. Cronbach’s *α* ranged from 0.810 to 0.861, composite reliability from 0.813 to 0.860, and average variance extracted from 0.473 to 0.593. The AVE for BI was 0.473, slightly below the conventional 0.50 threshold, whereas its composite reliability was 0.818 and its factor loadings ranged from 0.662 to 0.719. BI responses also showed moderate concentration at the upper end of the scale, with a mean top-two-box proportion of 71.4% and item skewness ranging from −0.90 to −0.77. All five response categories were represented, and no single category accounted for more than 50% of responses to any item. Discriminant validity was supported by the Fornell–Larcker criterion and HTMT values ranging from 0.324 to 0.784. Detailed measurement results are presented in Table 3 and Figure 1.

**Table 3:** Standardized factor loadings, reliability, and convergent validity.

Construct	Item	Standardized factor loading	Cronbach’s *α*	CR	AVE
Awareness of consequences (AC)	AC1	0.694	0.813	0.813	0.521
	AC2	0.757			
	AC3	0.673			
	AC4	0.760			
Ascription of responsibility (AR)	AR1	0.852	0.810	0.813	0.593
	AR2	0.744			
	AR3	0.707			
Personal norms (PN)	PN1	0.737	0.861	0.860	0.552
	PN2	0.776			
	PN3	0.755			
	PN4	0.745			
	PN5	0.700			
Behavioral intention (BI)	BI1	0.679	0.817	0.818	0.473
	BI2	0.677			
	BI3	0.662			
	BI4	0.719			
	BI5	0.701			

CR = composite reliability; AVE = average variance extracted. All standardized factor loadings were statistically significant.

### 4.3 Structural model and indirect associations

The fully sequential model (S1) was first tested, *χ2* = 138.225, df = 116. A six-path model (S2), which additionally included AC → PN, AC → BI, and AR → BI, fit significantly better than S1, *χ2* = 119.827, df = 113, Δ*χ2* = 18.398, Δdf = 3, *p* < 0.001. However, the paths from AC to PN (*β* = 0.004, 95% CI [−0.098, 0.108]) and from AR to BI (*β* = −0.023, 95% CI [−0.142, 0.091]) were not statistically significant, whereas the path from AC to BI was significant (*β* = 0.182, 95% CI [0.090, 0.277]). A more parsimonious partial mediation model (S3) was therefore tested after removing the two nonsignificant paths. S3 fit significantly better than S1, Δ*χ2* = 18.241, Δdf = 1, *p* < 0.001, and did not differ significantly from S2, Δ*χ2* = 0.157, Δdf = 2, *p* = 0.925. S3 also had the lowest AIC and BIC among the three models and was retained as the final model (Table 4).

**Table 4:** Comparison of structural models.

Model	*χ2*	*df*	*χ2*/*df*	CFI	TLI	RMSEA	SRMR	AIC	BIC
S1: Fully sequential mediated chain model (3 paths)	138.225	116	1.192	0.995	0.994	0.018	0.037	27104.709	27266.269
S2: Six-path model (all direct paths added)	119.827	113	1.060	0.998	0.998	0.010	0.025	27092.280	27266.938
S3: Final partial mediation model (4 paths)	119.984	115	1.043	0.999	0.999	0.009	0.025	27088.436	27254.362

S1 specified the fully sequential mediation pathway AC → AR → PN → BI. S2 additionally included the direct paths AC → PN, AC → BI, and AR → BI. S3 retained the AC → BI path and constrained the nonsignificant AC → PN and AR → BI paths to zero. AC = awareness of consequences; AR = ascription of responsibility; PN = personal norms; BI = behavioral intention; CFI = comparative fit index; TLI = Tucker–Lewis index; RMSEA = root mean square error of approximation; SRMR = standardized root mean square residual; AIC = Akaike information criterion; BIC = Bayesian information criterion.

**Table 5:** Path coefficients for the final structural model.

Structural path	*B*	SE	95% CI for *B*	β	C.R.	*p*
AC → AR	0.637	0.060	0.523, 0.758	0.522	10.650	<0.001
AR → PN	0.557	0.042	0.479, 0.643	0.617	13.182	<0.001
PN → BI	0.562	0.046	0.476, 0.655	0.727	12.153	<0.001
AC → BI	0.148	0.038	0.078, 0.227	0.173	3.923	<0.001

*B* = unstandardized path coefficient; SE = standard error; CI = confidence interval; *β* = standardized path coefficient; C.R. = critical ratio. The 95% confidence intervals are bias-corrected bootstrap confidence intervals based on 5,000 resamples. The final model explained 27.3% of the variance in AR (*R^2^* = 0.273), 38.0% in PN (*R^2^* = 0.380), and 64.0% in BI (*R^2^* = 0.640). AC = awareness of consequences; AR = ascription of responsibility; PN = personal norms; BI = behavioral intention.

The retained model included AC → AR, AR → PN, PN → BI, and AC → BI and showed a good fit, *χ2* = 119.984, df = 115, *p* = 0.357, CFI = 0.999, TLI = 0.999, RMSEA = 0.009, 90% CI [0.000, 0.023], and SRMR = 0.025. Awareness of consequences was positively associated with ascription of responsibility (*β* = 0.522, *p* < 0.001), ascription of responsibility was positively associated with personal norms (*β* = 0.617, *p* < 0.001), and personal norms were positively associated with behavioral intention (*β* = 0.727, *p* < 0.001). Awareness of consequences was also directly associated with behavioral intention (*β* = 0.173, *p* < 0.001). The model explained 27.3% of the variance in ascription of responsibility, 38.0% of the variance in personal norms, and 64.0% of the variance in behavioral intention.

Bias-corrected bootstrap analysis based on 5,000 resamples showed significant indirect associations for AC → AR → PN (standardized estimate = 0.322, 95% CI [0.263, 0.381]), AR → PN → BI (standardized estimate = 0.449, 95% CI [0.386, 0.511]), and AC → AR → PN → BI (standardized estimate = 0.234, 95% CI [0.188, 0.282]). The standardized direct association between AC and BI was 0.173 (95% CI [0.093, 0.255]), and the standardized total association was 0.407 (95% CI [0.322, 0.487]). The observed direct and indirect associations were consistent with H1–H6. The significant sequential indirect association, together with the remaining direct association between AC and BI, was consistent with a partial mediation pattern. Complete model comparisons and estimates are presented in Tables 4–6 and Figure 2.

**Table 6:** Direct, indirect, and total effects in the final structural model.

Effect	*B*	Bootstrap SE	95% CI for *B*	*β*	95% CI for *β*
Indirect effect: AC → AR → PN	0.355	0.041	0.281, 0.442	0.322	0.263, 0.381
Indirect effect: AR → PN → BI	0.313	0.031	0.257, 0.376	0.449	0.386, 0.511
Sequential and total indirect effect: AC → AR → PN → BI	0.200	0.026	0.154, 0.255	0.234	0.188, 0.282
Direct effect: AC → BI	0.148	0.038	0.078, 0.227	0.173	0.093, 0.255
Total effect: AC → BI	0.347	0.045	0.265, 0.441	0.407	0.322, 0.487

*B* = unstandardized effect; SE = standard error; CI = confidence interval; *β* = standardized effect. Bootstrap standard errors and bias-corrected 95% confidence intervals were based on 5,000 resamples. Unstandardized and standardized effects and their confidence intervals are reported on separate scales. Because the final model included only one indirect pathway from AC to BI, the sequential indirect effect was equivalent to the total indirect effect. The significant sequential indirect association and the remaining direct association were consistent with a partial mediation pattern; these associations should not be interpreted as causal effects. AC = awareness of consequences; AR = ascription of responsibility; PN = personal norms; BI = behavioral intention.

**Figure 2:**
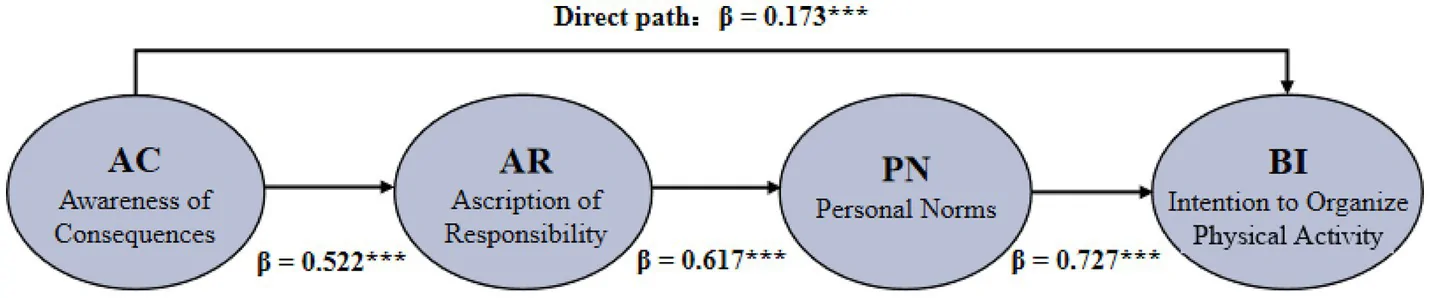
Structural model of preschool teachers’ intention to organize physical activity. AC = awareness of consequences; AR = ascription of responsibility; PN = personal norms; BI = behavioral intention. Standardized path coefficients are presented. ^***^p < 0.001.

### 4.4 Robustness of the structural model

The retained model was further examined using a non-nested alternative structure, a polychoric-correlation refit, and a covariate-adjusted model. The alternative model showed poorer fit than S3, whereas the principal path estimates obtained from the polychoric-correlation and covariate-adjusted analyses were similar in direction and magnitude to those from the primary ML analysis. The standardized sequential indirect estimate was 0.237 in the polychoric-correlation analysis and 0.236 (95% CI [0.193, 0.286]) after covariate adjustment, compared with 0.234 (95% CI [0.188, 0.282]) in the primary model. Full results are presented in Supplementary Table S2.

## 5 Discussion

### 5.1 Key findings and interpretation

Grounded in NAM, the present study examined the associations among responsibility-related normative factors and preschool teachers’ intention to organize physical activity. All hypothesized relationships were supported, and the proposed structural model accounted for a substantial proportion of the variance in behavioral intention (Schwartz, 1977; de Groot and Steg, 2009). These findings provide empirical support for the applicability of NAM to understanding preschool teachers’ intention to organize physical activity. In particular, personal norms emerged as the normative factor most closely associated with behavioral intention, highlighting the importance of teachers’ personal sense of moral obligation in shaping their intention to provide physical activity opportunities.

Within this responsibility-related pattern, personal norms were most closely associated with preschool teachers’ intention to organize physical activity. Organizing physical activity requires teachers to safeguard children’s activity time, prepare appropriate activity content, sustain activities under constrained conditions, and adjust activity arrangements according to children’s needs. Teachers’ intention may therefore depend not only on whether they recognize the value of physical activity, but also on whether they personally feel obliged to provide children with adequate opportunities to be active. Moral norms, perceived behavioral control, and social expectations have all been associated with childcare workers’ intention to involve preschool children in physical activity (Gagné and Harnois, 2014). The present findings further suggest that personal norms are closely linked to teachers’ sense of personal responsibility for providing physical activity opportunities.

The positive association between awareness of consequences and ascription of responsibility indicates that recognizing the developmental risks associated with insufficient physical activity and regarding the management of those risks as one’s own responsibility are related but distinct judgments. Preschool teachers may fully recognize that insufficient physical activity can adversely affect children’s physical health, motor development, emotional functioning, and peer interactions, yet still regard preschool administrators, families, or other relevant parties as primarily responsible for ensuring adequate activity opportunities. Responsibility may become particularly diffuse when decisions concerning schedules, staffing, facilities, and safety management are made beyond the classroom level (Copeland et al., 2012; Jerebine et al., 2024; Tonge et al., 2024).

Ascription of responsibility was also positively associated with personal norms. Professional responsibilities may be formally prescribed through curriculum requirements, institutional policies, and occupational standards, whereas personal norms emphasize whether teachers personally accept these responsibilities as obligations of their own. Teachers may acknowledge that organizing physical activity is part of their professional role, but may not prioritize it when it conflicts with curricular demands, administrative duties, safety concerns, or limited resources. Teachers’ sense of personal responsibility is conceptually distinct from both formal accountability and teachers’ self-efficacy beliefs (Lauermann and Karabenick, 2013; Matteucci et al., 2017). The present findings suggest that teachers who more clearly regarded children’s physical activity as their own responsibility were also more likely to experience a personal obligation to act.

The indirect association between awareness of consequences and personal norms through ascription of responsibility suggests that the relationship between teachers’ recognition of developmental consequences and their sense of personal obligation was partly reflected in how they understood their own responsibility. When teachers continue to assign responsibility to other individuals or institutional levels, simply knowing that insufficient physical activity may be harmful may not be sufficient to generate a personal obligation to intervene. Clarifying how teachers’ everyday educational decisions shape children’s opportunities to be physically active may therefore help connect knowledge of child development with personally accepted responsibility (Copeland et al., 2012; Jerebine et al., 2024).

The association between ascription of responsibility and behavioral intention was also partly reflected through personal norms. Acknowledging responsibility does not necessarily mean that teachers will intend to change their educational practice. When such responsibility is personally accepted as an obligation to safeguard children’s activity time, improve teaching practice, and respond to inadequate physical activity provision, responsibility judgments may become more closely connected with behavioral intention. Personal norms may therefore represent an important link between general judgments of responsibility and intended professional action (Gagné and Harnois, 2014; Lauermann and Karabenick, 2013).

At the same time, the remaining direct association between awareness of consequences and behavioral intention indicates that ascription of responsibility and personal norms do not fully account for how consequence awareness is related to intended practice. Awareness of developmental risks may increase teachers’ perceptions of the importance or urgency of physical activity. It may also operate alongside behavioral attitudes, professional self-efficacy, perceived behavioral control, and support from preschool directors, colleagues, and parents (Gagné and Harnois, 2014; Bruijns et al., 2021; Bourke et al., 2024). These factors may help explain why teachers who more clearly recognize the consequences of insufficient physical activity continue to report stronger behavioral intentions even after responsibility attribution and personal obligation are considered. Because these factors were not directly measured in the present study, their potential explanatory roles require further empirical examination.

The present findings therefore describe a responsibility-related motivational dimension of preschool teachers’ intention to organize physical activity rather than a complete explanation of their actual educational practice. Whether physical activity is implemented in practice also depends on teachers’ professional knowledge, practical skills, available time and space, staffing arrangements, competing curricular priorities, and administrative support (Copeland et al., 2012; Jerebine et al., 2024; Tonge et al., 2024; Wright et al., 2024). Teachers may recognize the consequences of insufficient physical activity, accept responsibility, and form an intention to act while still lacking the resources, authority, or practical capability required to organize physical activity regularly. Responsibility-related interventions should therefore be combined with professional training and organizational support rather than placing the burden of implementation entirely on individual teachers (Bourke et al., 2024).

### 5.2 Theoretical implications

By situating personal norms within a responsibility-related structure comprising awareness of consequences and ascription of responsibility, this study further clarifies their role in explaining preschool teachers’ intention to organize physical activity. Previous research on childcare workers identified moral norm as an important correlate of behavioral intention but did not examine its position within a broader responsibility-related structure involving consequence awareness and responsibility attribution (Gagné and Harnois, 2014). The present findings indicate that personal norms are closely related to how teachers understand the consequences of insufficient physical activity and how they evaluate their own responsibility for addressing them. Conceptually, this framework also helps differentiate externally prescribed professional expectations, teachers’ judgments of personal responsibility, and personally endorsed obligations to act.

### 5.3 Practical implications

The findings have implications for both initial teacher education and continuing professional development. In initial teacher education, physical activity can be incorporated more explicitly into training on child development and everyday teaching practice. Future teachers need opportunities to understand how insufficient physical activity may affect children’s development and how decisions about scheduling, activity content, indoor and outdoor play, and instructional support influence children’s opportunities to be active. Linking this knowledge with practical experience may help teachers develop a clearer understanding of their role in supporting children’s physical activity.

For practicing teachers, continuing professional development can focus more closely on the organization of physical activity in everyday preschool routines. Training can help teachers identify practical ways to maintain activity opportunities when time, space, staffing, or safety concerns limit what can be provided. It can also help teachers distinguish between aspects of physical activity provision that can be managed within classroom practice and those that require coordination with colleagues or administrators. Sustained physical activity provision depends on both teachers’ willingness to act and the conditions available to support their practice, including curriculum arrangements, access to space, staffing, and administrative support.

### 5.4 Limitations and future research

Several limitations should be acknowledged. Because the study employed a cross-sectional design, the temporal ordering of awareness of consequences, ascription of responsibility, personal norms, and behavioral intention cannot be determined. The observed sequential indirect association was consistent with the ordering proposed by NAM. Establishing temporal and causal relationships will require longitudinal or experimental evidence. The study also focused on teachers’ intention to organize physical activity rather than their actual behavior. It therefore remains unclear whether stronger intention is associated with more consistent organization of physical activity in practice or with higher levels of children’s physical activity. In addition, all variables were assessed through teacher self-reports and may therefore have been affected by social desirability and common method bias. Convenience sampling and recruitment through kindergarten contacts and online preschool teacher groups may limit sample representativeness and the generalizability of the findings across regions and educational contexts.

Future research should examine how these constructs are related over time to determine whether stable temporal relationships exist among awareness of consequences, ascription of responsibility, and personal norms. Experimental designs may also be used to investigate how different responsibility-related messages or situational cues influence teachers’ normative judgments. Further research should move beyond behavioral intention by combining questionnaire data with classroom observations, activity records, and objective measures of children’s physical activity. Such designs would make it possible to examine whether stronger personal norms are associated with more consistent organization of physical activity in practice and how these practices are associated with teachers’ professional competence, available time and space, and administrative support. Replicating the model in samples of preschool teachers recruited using more systematic sampling approaches across diverse regional, institutional, and cultural contexts would also help determine the stability and generalizability of the responsibility-related structure identified in the present study.

## 6 Conclusion

Drawing on NAM, this study examined the responsibility-related normative relationships underlying preschool teachers’ intention to organize physical activity. The findings suggest that awareness of the adverse consequences of insufficient physical activity alone may not fully account for teachers’ intention to act. Teachers reported a stronger intention when they regarded addressing these consequences as their own responsibility and personally endorsed an obligation to act. Efforts to promote physical activity in preschool settings should help teachers clarify their responsibilities and provide the professional and organizational support needed to create regular physical activity opportunities. In this context, initial teacher education and continuing professional development can serve as practical contexts for linking teachers’ understanding of children’s developmental needs and professional responsibilities with strategies for systematically organizing physical activity.

## Data Availability

The raw data supporting the conclusions of this article will be made available by the authors, without undue reservation.
