# Supplementary material for: Preschool teachers’ intention to organize physical activity: an application of the norm activation model
Source: Front Psychol. 2026 Oct 1;17:1971172. doi: 10.3389/fpsyg.2026.1971172 (PMC13669177; doi:10.3389/fpsyg.2026.1971172)
Supplement: Supplementary file 1 [file Supplementary_file_1.DOCX]

**Preschool Teachers’ Intentions to Organize Physical Activity: An Application of the Norm Activation Model**

# Supplementary Material

## Supplementary Table S1. Administered measurement items

The table lists the 17 Chinese items administered in the study, English translations, response anchors, scoring direction, and the behavioral target or time frame represented by each item.

Note. All items used a five-point response scale from 1 (completely disagree) to 5 (completely agree). All items were positively keyed; no reverse-worded item was administered.

| **Construct** | **Item** | **English translation** | **Response scale** | **Reverse scored** | **Behavioral target/time frame** |
| --- | --- | --- | --- | --- | --- |
| AC | AC1 | Reducing physical activity time is likely to increase the possibility of declines in children's physical fitness (e.g., becoming prone to illness). | 1 = completely disagree; 2 = somewhat disagree; 3 = uncertain; 4 = somewhat agree; 5 = completely agree. | No; positively keyed. | No fixed time frame. |
| AC | AC2 | A lack of physical activity may delay children's motor development, such as weakening their ability to run and jump. | 1 = completely disagree; 2 = somewhat disagree; 3 = uncertain; 4 = somewhat agree; 5 = completely agree. | No; positively keyed. | No fixed time frame. |
| AC | AC3 | Failing to organize physical activities may make children more likely to experience emotional problems, such as irritability or difficulty concentrating. | 1 = completely disagree; 2 = somewhat disagree; 3 = uncertain; 4 = somewhat agree; 5 = completely agree. | No; positively keyed. | No fixed time frame. |
| AC | AC4 | A prolonged lack of physical activity may affect children's ability to interact and cooperate with peers. | 1 = completely disagree; 2 = somewhat disagree; 3 = uncertain; 4 = somewhat agree; 5 = completely agree. | No; positively keyed. | Long-term exposure. |
| AR | AR1 | Promoting children's physical health is an important responsibility of mine as a preschool teacher. | 1 = completely disagree; 2 = somewhat disagree; 3 = uncertain; 4 = somewhat agree; 5 = completely agree. | No; positively keyed. | No fixed time frame. |
| AR | AR2 | Ensuring children's safety during physical activities requires me to make preventive preparations and prepare appropriate responses in advance. | 1 = completely disagree; 2 = somewhat disagree; 3 = uncertain; 4 = somewhat agree; 5 = completely agree. | No; positively keyed. | Advance preparation. |
| AR | AR3 | It is my responsibility to pay attention to each child's participation and development during physical activities. | 1 = completely disagree; 2 = somewhat disagree; 3 = uncertain; 4 = somewhat agree; 5 = completely agree. | No; positively keyed. | During physical activities. |
| PN | PN1 | Even when I am busy, I feel obliged to ensure children's daily physical activity time. | 1 = completely disagree; 2 = somewhat disagree; 3 = uncertain; 4 = somewhat agree; 5 = completely agree. | No; positively keyed. | Daily target. |
| PN | PN2 | When I notice that children have weak physical fitness, I will take the initiative to increase targeted physical activity content. | 1 = completely disagree; 2 = somewhat disagree; 3 = uncertain; 4 = somewhat agree; 5 = completely agree. | No; positively keyed. | Conditional on weak fitness. |
| PN | PN3 | I believe that I have an obligation to take the initiative to learn new knowledge and methods related to preschool physical education. | 1 = completely disagree; 2 = somewhat disagree; 3 = uncertain; 4 = somewhat agree; 5 = completely agree. | No; positively keyed. | No fixed time frame. |
| PN | PN4 | If my kindergarten lacks physical activity resources, I will try to create conditions for activities, for example by using recycled materials. | 1 = completely disagree; 2 = somewhat disagree; 3 = uncertain; 4 = somewhat agree; 5 = completely agree. | No; positively keyed. | Conditional on resource constraints. |
| PN | PN5 | If I fail to ensure children's physical activity time, I feel responsible for making improvements. | 1 = completely disagree; 2 = somewhat disagree; 3 = uncertain; 4 = somewhat agree; 5 = completely agree. | No; positively keyed. | Conditional on failing to ensure activity time. |
| BI | BI1 | In the coming semester, I plan to increase the frequency with which I organize physical activities (e.g., from twice to four times per week). | 1 = completely disagree; 2 = somewhat disagree; 3 = uncertain; 4 = somewhat agree; 5 = completely agree. | No; positively keyed. | Coming semester; example changes frequency from twice to four times weekly. |
| BI | BI2 | I am willing to spend more time designing a wider variety of physical activities, such as outdoor games, sensory integration training, and ball games. | 1 = completely disagree; 2 = somewhat disagree; 3 = uncertain; 4 = somewhat agree; 5 = completely agree. | No; positively keyed. | Future willingness; no fixed interval. |
| BI | BI3 | I will actively register for training or teaching-research activities related to preschool physical education. | 1 = completely disagree; 2 = somewhat disagree; 3 = uncertain; 4 = somewhat agree; 5 = completely agree. | No; positively keyed. | Future action; no fixed interval. |
| BI | BI4 | Even when I encounter difficulties, such as limited space or low child cooperation, I will continue to organize physical activities. | 1 = completely disagree; 2 = somewhat disagree; 3 = uncertain; 4 = somewhat agree; 5 = completely agree. | No; positively keyed. | Conditional persistence under difficulties. |
| BI | BI5 | I will regularly reflect on the effectiveness of physical activities and adjust activity plans according to children's feedback. | 1 = completely disagree; 2 = somewhat disagree; 3 = uncertain; 4 = somewhat agree; 5 = completely agree. | No; positively keyed. | Regular reflection; interval unspecified. |

**Supplementary Table S2. Summary of Primary and Sensitivity Analyses**

| **Analysis/model** | **Estimator** | **Specification** | **N** | **Focal standardized paths (β)** | **Sequential indirect β** | **Fit indices** | **AIC / BIC** | **Main conclusion** |
| --- | --- | --- | --- | --- | --- | --- | --- | --- |
| S1: fully sequential model | ML | AC→AR→PN→BI; AC→BI fixed to 0 | 582 | AC→AR=.527; AR→PN=.630; PN→BI=.791 | .262, 95% BC CI [.211, .318] | χ²=138.225, df=116, CFI=.995, TLI=.994, RMSEA=.018, SRMR=.037 | 27104.709 / 27266.269 | Fit was good, but adding AC→BI improved fit. |
| S2: six-path model | ML | S1 plus AC→PN, AC→BI, and AR→BI | 582 | AC→AR=.522; AR→PN=.616; PN→BI=.740; AC→BI=.182; AC→PN=.004; AR→BI=−.023 | .238, 95% BC CI [.179, .309] | χ²=119.827, df=113, CFI=.998, TLI=.998, RMSEA=.010, SRMR=.025 | 27092.280 / 27266.938 | Only AC→BI was significant among the added direct paths; the sequential indirect association remained significant. |
| S3: retained partial mediation model | ML | AC→AR→PN→BI plus AC→BI | 582 | AC→AR=.522; AR→PN=.617; PN→BI=.727; AC→BI=.173 | .234, 95% BC CI [.188, .282] | χ²=119.984, df=115, CFI=.999, TLI=.999, RMSEA=.009, SRMR=.025 | 27088.436 / 27254.362 | Retained as the more parsimonious partial mediation model. |
| S02: parallel/common-antecedent alternative | ML | AC→AR, AC→PN, AR→BI, PN→BI | 582 | AC→AR=.569; AC→PN=.402; AR→BI=.148; PN→BI=.733 | NA (no sequential pathway) | χ²=252.909, df=115, CFI=.966, TLI=.960, RMSEA=.045, SRMR=.084 | 27221.591 / 27387.516 | Fit was poorer than S3; the comparison does not establish causal ordering. |
| S04: ordinal-correlation sensitivity | ML fitted to a polychoric matrix; not WLSMV | S3 refitted to polychoric correlations | 582 | AC→AR=.524; AR→PN=.625; PN→BI=.724; AC→BI=.181 | .237 (point estimate; CI not estimated) | χ²=182.665, df=115, CFI=.986, TLI=.984, RMSEA=.032, SRMR=.028 | NA / NA | Core path magnitudes and the indirect point estimate were close to the primary ML results; estimator-specific inference was not tested. |
| S05: BI item-distribution diagnostic | Descriptive | Top-two-box proportions and item skewness | 582 | NA | NA | BI top-two-box=71.4%; skewness −.90 to −.77 | NA / NA | Moderate concentration at the upper end of the response scale; all five BI items were retained. |
| S08: covariate-adjusted model | ML with measured covariates | S3 adjusted for PE-related educational background, full-time PE-teacher provision, and kindergarten type | 582 | AC→AR=.520; AR→PN=.615; PN→BI=.737; AC→BI=.172 | .236, 95% BC CI [.193, .286] | χ²=179.173, df=154, χ²/df=1.163, RMSEA=.017, SRMR=.026; CFI/TLI=NR | 29590.676 / 29835.198 | Core paths changed by .001–.010 and the sequential indirect association remained significant; unmeasured confounding remains possible. |
| S07: marker/method-factor analyses | Not conducted | No theoretically unrelated marker construct or social-desirability scale was administered | NA | NA | NA | NA | NA / NA | Harman’s test does not rule out common method bias. |

Note. ML = maximum likelihood; BC CI = bias-corrected confidence interval; NR = not reported; NA = not applicable. All reported BC confidence intervals for S1, S2, S3, and S08 were based on 5,000 bootstrap resamples. The S04 sequential indirect effect is a point estimate only; no bootstrap confidence interval was estimated. S04 was an exploratory ML refit to a polychoric correlation matrix, not a complete WLSMV analysis, and its fit indices are not treated as WLSMV-robust statistics. AIC/BIC comparisons are interpreted only for models estimated with the same data, observed-variable set, and estimator; the S08 information criteria are therefore not compared with S1–S3. No χ² difference test was used for the non-nested S02 alternative. S07 was not conducted because the questionnaire contained no suitable marker construct or dedicated social-desirability measure.
